# Evaluation of Silk Biomaterials in Combination with Extracellular Matrix Coatings for Bladder Tissue Engineering with Primary and Pluripotent Cells

**DOI:** 10.1371/journal.pone.0056237

**Published:** 2013-02-07

**Authors:** Debra Franck, Eun Seok Gil, Rosalyn M. Adam, David L. Kaplan, Yeun Goo Chung, Carlos R. Estrada, Joshua R. Mauney

**Affiliations:** 1 Department of Urology, Urological Diseases Research Center, Boston Children's Hospital, Boston, Massachusetts, United States of America; 2 Department of Surgery, Harvard Medical School, Boston, Massachusetts, United States of America; 3 Department of Biomedical Engineering, Tufts University, Medford, Massachusetts, United States of America; Université de Technologie de Compiègne, France

## Abstract

Silk-based biomaterials in combination with extracellular matrix (ECM) coatings were assessed as templates for cell-seeded bladder tissue engineering approaches. Two structurally diverse groups of silk scaffolds were produced by a gel spinning process and consisted of either smooth, compact multi-laminates (Group 1) or rough, porous lamellar-like sheets (Group 2). Scaffolds alone or coated with collagen types I or IV or fibronectin were assessed independently for their ability to support attachment, proliferation, and differentiation of primary cell lines including human bladder smooth muscle cells (SMC) and urothelial cells as well as pluripotent cell populations, such as murine embryonic stem cells (ESC) and induced pluripotent stem (iPS) cells. AlamarBlue evaluations revealed that fibronectin-coated Group 2 scaffolds promoted the highest degree of primary SMC and urothelial cell attachment in comparison to uncoated Group 2 controls and all Group 1 scaffold variants. Real time RT-PCR and immunohistochemical (IHC) analyses demonstrated that both fibronectin-coated silk groups were permissive for SMC contractile differentiation as determined by significant upregulation of α-actin and SM22α mRNA and protein expression levels following TGFβ1 stimulation. Prominent expression of epithelial differentiation markers, cytokeratins, was observed in urothelial cells cultured on both control and fibronectin-coated groups following IHC analysis. Evaluation of silk matrices for ESC and iPS cell attachment by alamarBlue showed that fibronectin-coated Group 2 scaffolds promoted the highest levels in comparison to all other scaffold formulations. In addition, real time RT-PCR and IHC analyses showed that fibronectin-coated Group 2 scaffolds facilitated ESC and iPS cell differentiation toward both urothelial and smooth muscle lineages in response to *all trans* retinoic acid as assessed by induction of uroplakin and contractile gene and protein expression. These results demonstrate that silk scaffolds support primary and pluripotent cell responses pertinent to bladder tissue engineering and that scaffold morphology and fibronectin coatings influence these processes.

## Introduction

Surgical management of a variety of bladder disorders including neurogenic bladder, posterior urethral valves, and bladder exstrophy frequently requires augmentation cystoplasty in order to prevent renal damage and incontinence from increased urinary storage and voiding pressures [1]. Currently, gastrointestinal segments represent the primary option for bladder reconstructive procedures despite numerous complications including chronic urinary tract infection, stone formation, graft rupture, as well as secondary malignancies [2]. Previous studies in bladder tissue engineering have investigated both natural and synthetic scaffolds either alone or seeded with autologous bladder cell populations as alternative strategies for defect repair or neobladder reconstruction [3]. Conventional scaffolds such as acellular bladder matrix (ABM), poly-glycolic acid (PGA), and small intestinal submucosa (SIS) have been reported to support bladder tissue regeneration in various animal models [4]–[6] and in some cases short-term clinical trials [7], [8]. However these matrices are frequently associated with various deleterious side effects including implant contracture, calcification, and fibrosis and therefore may be restricted in their long-term clinical potential [9]–[11].

Biomaterials derived from *Bombyx mori* silk fibroin represent an emerging platform for bladder tissue engineering due to their mechanical robustness [12], processing plasticity [13], and biodegradability [14]. In particular, the process of gel spinning allows for the formation of 3-D biomaterials with selective structural and mechanical properties through controlled variations in winding and post-winding fabrication parameters [15]–[17]. Our previous results have shown that acellular silk scaffolds were capable of supporting murine bladder augmentation wherein they displayed significant advantages in comparison to PGA and SIS matrices including superior tissue regeneration, reduced inflammatory reactions, and improved functional performance [16]. In addition, we reported that manipulation of silk matrix properties can influence the extent of *in vivo* scaffold degradation and host tissue integration [17]. These studies reveal that the silk gel spinning process represents a tunable system for understanding the ability of specific biomaterial characteristics to support reconstitution of lower urinary tract defects.

Large animal studies of bladder augmentation have highlighted several limitations associated with the use of acellular scaffolds for defect consolidation [18], [19]. These matrices rely on the capacity of host cell populations to migrate, expand, and differentiate throughout the wound site in order to promote *de novo* tissue formation and restoration of organ function. In a non-diseased porcine model, Brown and colleagues observed that host tissue integration was restricted to the periphery of SIS grafts (46 cm^2^), while central regions of the implant failed to support organized smooth muscle bundles or urothelial maturation [18]. These results suggest that acellular biomaterials may be hampered in their ability to heal defects of clinically relevant size due to a lack of host cell infiltration. This issue can be further compounded by disease pathology, wherein aberrations in host cell functionality may restrict their ability to populate and repair defect sites. Indeed, a study by the Kaefer group demonstrated that while augmentation of healthy porcine bladders with an acellular dermal biomatrix resulted in functional regeneration of small scale defects, similar experiments in an obstructed bladder model failed to show favorable results [19].

Cell seeding of biomaterials prior to implantation has been demonstrated as a viable approach for enhancing the repair efficacy of acellular scaffolds. Atala and colleagues reported that PGA matrices seeded with primary smooth muscle cells (SMC) and urothelial cells improved the extent of bladder tissue regeneration over non-seeded controls [5]. The feasibility of tissue engineered constructs seeded with autologous primary cells has also been validated in short term clinical trials of bladder augmentation [7]. Unfortunately, tissue-specific primary cells may be limited for therapeutic use in some patients due to a lack of availability or diseased phenotype [20], [21]. To circumvent these issues, researchers have investigated the utility of multi-potent adult stem cells for bladder reconstruction including both bone marrow and adipose-derived mesenchymal stem cells (MSC) [22], [23]. These reports have shown that scaffold-seeded MSC populations can contribute to bladder smooth muscle regeneration *in vivo*; however their capacity to undergo urothelial engraftment has not been observed. MSC have been reported to progress toward a urothelial lineage *in vitro* under selective culture conditions [24], [25], however these cell types may be restricted in their *in vivo* potential to differentiate into mature urothelium. These studies highlight the need to evaluate other cell sources for bladder tissue repair.

Pluripotent populations such as embryonic stem cells (ESC) and induced pluripotent stem (iPS) cells may fulfill the need for alternative cell sources for bladder regeneration. ESC are derived from the inner cell mass of blastocysts and have the potential for the production of patient-specific tissues either through stem cell banking or via somatic cell nuclear transfer [26]. iPS cells are reprogrammed, terminally differentiated somatic cells which have acquired ESC-like properties following ectopic expression of various pluripotency transcription factors [27], [28]. Unlike MSC which undergo senescence following prolonged *ex vivo* cultivation [29], these cell lines can be theoretically expanded for indefinite periods [27], [30], thus providing large amounts of cells for tissue repair. In comparison to multi-potent MSC, the pluripotent nature of ESC and iPS cells also endows them with the capacity to differentiate into a greater assortment of potential lineages for the creation of tissue engineered constructs [31]. In addition, we have previously reported the ability of murine ESC to undergo both SMC and urothelial differentiation *in vitro* [32], while iPS cells have been shown to acquire features of contractile SMC [33]. Development of strategies which can deliver these pluripotent cell lines into defect sites as well as promote their lineage-specific differentiation is desired to generate functional bladder tissue and avoid unwanted teratoma formation.

Biomaterial configurations possessing microenviromental features appropriate to enable attachment, proliferation, and differentiation of both primary and pluripotent cells have the advantage of providing clinicians flexibility in using available cell sources with a common scaffold template for bladder tissue engineering. Therefore, the goal in the present study was to assess the potential of gel spun silk scaffolds to support these processes in primary SMC and urothelial cell lines as well as ESC and iPS cells. In addition, since previous investigations have demonstrated that functionalization of silk biomaterials with tissue-specific extracellular matrix (ECM) proteins and motifs enhances their overall biocompatibility [34], [35], we also determined the effect of different ECM protein coatings on the ability of silk matrices to support cell responses in parallel.

## Materials and Methods

### Ethics Statement

Bombyx mori silkworm cocoons were obtained from a commercial supplier (Tajima Shoji Co., Yokohama, Japan) and therefore no specific field studies were performed for their acquisition.

### Biomaterials

Aqueous silk fibroin solutions were prepared from *Bombyx mori* silkworm cocoons using previously described procedures [12]. Silk matrices were formed using a published gel spinning technique [15]. Tubular scaffolds were produced by spinning concentrated silk solutions [25–30% (w/v), 0.5 ml/scaffold] onto a rotating (200 rpm) and axially reciprocating mandrel (6 mm in diameter) using a custom gel spinning platform and program. Two groups of matrices previously shown to support murine bladder augmentation, but with different structural and mechanical properties, were generated with various winding and post-winding conditions [16], [17]. Group 1 scaffolds were spun with an axial slew rate (ASR) of 2 mm/sec followed by treatment with methanol. Group 2 scaffolds were composed of ∼0.4 ml of silk solution spun at an ASR of 40 mm/sec followed by ∼0.1 ml spun at 2 mm/sec in order to consolidate gaps between the resultant silk fibers. This matrix group was then subjected to lyophilization and subsequent methanol treatment. The tubes were then removed from the mandrel after briefly soaking in a surfactant solution. Matrices were bisected along their central axis, cut into individual squares (5×5 mm^2^), sterilized in 70% ethanol, and rinsed in phosphate buffered saline (PBS) overnight. To facilitate ECM protein coatings, collagen type I (from rat tail, cat.# 1179179, Roche Applied Science, Indianapolis, IN), collagen type IV (from human placenta, cat.# C7521, Sigma Aldrich, St. Louis, MO), and fibronectin (from bovine plasma, cat.# F4759, Sigma Aldrich) were prepared as sterile aqueous solutions (0.1 mg/ml) and independently incubated with silk scaffolds for 1 h at 37°C followed by 1 h of drying in a laminar flow hood at room temperature. Uncoated controls were prepared in parallel with incubation in PBS.

### Scanning electron microscopy (SEM)

Structural analysis of uncoated matrix groups was performed in order to assess differences in scaffold morphology. Matrix samples were sputter coated with gold and imaged using a Hitachi S-520 Scanning Electron Microscope (Hitachi, Tokyo, Japan).

### Bladder Smooth Muscle Cells

Human primary SMC, isolated from the urinary bladder (passage 3), were obtained commercially (Lonza, Walkersville, MD) and expanded for 3 additional passages on 185 cm^2^ tissue culture plates in Smooth Muscle Growth Medium-2 (SmGM-2, Lonza) according to the manufacturer's instructions. SmGM-2 consists of a proprietary Smooth Muscle Basal Medium (SmBM) containing 5% fetal calf serum (FCS), epidermal growth factor (EGF), insulin, basic fibroblast growth factor (bFGF), and gentamicin/amphotericin-B. For cell attachment and proliferation analyses, SMC (passage 6) were seeded on uncoated or ECM-coated matrices (10^6^ cells/scaffold) in SmGM-2 and cultured for up to 7 d. Medium exchange was carried out every 3 d. To induce contractile differentiation, SMC were seeded on matrices as described above, serum depleted for 24 h in SmBM containing 0.5% FCS, and subsequently exposed to 2.5 ng/ml TGFβ-1 (R&D Systems, Minneapolis, MN) for 24 h. Control cultures were maintained in parallel in SmGM-2 for 3 d.

### Urothelial Cells

A previously described non-transformed human urothelial cell line, TRT-HU1, derived from cadaveric bladder biopsies [36] was expanded to passage 13 on 185 cm^2^ tissue culture plates in basal medium consisting of Dulbecco's modified Eagle's medium (DMEM, Invitrogen, Carlsbad, CA) supplemented with 15% FCS (Invitrogen), 2 mM L-glutamine (Invitrogen), 100 units/ml penicillin (Invitrogen), 100 µg/ml streptomycin (Invitrogen), 0.1 mM nonessential amino acids (Invitrogen), and 1 mM 1-thioglycerol (Sigma Aldrich). Urothelial cells were seeded on uncoated or ECM-coated matrices (10^6^ cells/scaffold) and cultured in basal medium for up to 9 d to assess cell attachment, extent of proliferation, and differentiation status. Medium exchange was performed every 3 d.

### Embryonic and Induced Pluripotent Stem Cells

Murine ESC (C57BL/6 line, American Type Culture Collection, Manassas, VA) and iPS cells (T1b line, Stemgent, Cambridge, MA) were expanded on γ-irradiated murine embryonic fibroblasts (MEF) in DMEM supplemented with 15% FCS, 100 units/ml penicillin, 100 µg/ml streptomycin, 0.1 mM nonessential amino acids, 2 mM L-glutamine, 0.1 µM 2-mercaptoethanol (Millipore, Billerica, MA), and 10^3^ units/ml leukemia inhibitory factor (LIF, Millipore). Following expansion, pluripotency marker (OCT4) expression was assessed by immunocytochemical (ICC) analysis. Briefly, cultures were fixed with 10% neutral-buffered formalin and OCT4 expression was detected following incubation with primary antibody (anti-OCT4, Cell Signaling Technology, Danvers, MA, cat.# 2840, 1∶200 dilution) and species-matched Cy3-conjugated secondary antibody (Millipore). Nuclei were counterstained with 4′, 6-diamidino-2-phenyllindole (DAPI) and cultures were visualized as described below. To induce *in vitro* differentiation of bladder cell phenotypes, feeder-depleted ESC and iPS cells were seeded on uncoated or ECM-coated silk scaffolds (10^6^ cells/scaffold) in basal medium supplemented with *all trans* retinoic acid (RA, Sigma-Aldrich) [10 µM] and cultured for up to 14 d with daily medium changes as previously described [32]. Spontaneously differentiating controls in the absence of RA were maintained in parallel.

### Cell Attachment and Proliferation Analyses

The relative number of metabolically active cells following 24 h of cell seeding and over the course of cultivation on uncoated and ECM-coated silk matrices was determined by the alamarBlue assay (Invitrogen) according to the manufacturer's instructions. Briefly, scaffolds seeded with primary and pluripotent cell lines were incubated in their respective culture medium supplemented with 10% alamarBlue reagent for 2 h at 37°C with 5% CO_2_. Post reaction medium aliquots (100 µl) were transferred to 96 well plates and quantified for fluorescence intensity within a FLUOstar Omega plate reader (BMG Labtech Inc., Durham, NC) using an excitation wavelength of 560 nm and an emission wavelength of 590 nm. Non-seeded matrices were screened in parallel as background controls. Relative cell numbers were calculated as the degree of relative fluorescence units (FU) per construct.

### mRNA Analyses

Total RNA was extracted from seeded scaffolds according to the single step acid-phenol guanidinium method [37] using Trizol reagent (Invitrogen). mRNA was enriched from total RNA using the RNeasy kit (Qiagen Inc., Valencia, CA) and cDNAs were synthesized using the High-Capacity cDNA Transcription kit (Applied Biosystems, Foster City, CA) following the manufacturer's instructions. Real time RT-PCR reactions were performed and analyzed using the Applied Biosystems StepOnePlus™ Real time PCR Detection System and StepOne Software (version 2.1). cDNA samples were assessed for genes of interest and the housekeeping gene, GAPDH, in independent reactions using the Taqman Universal PCR master mix in combination with commercially available primers and probes consisting of Assays-on-Demand™ Gene Expression kits (Applied Biosystems) following the manufacturer's instructions. Expression kits included: murine: uroplakin (UP) 1A, Mm01176597_g1; UP1B, Mm00769504_m1 UP2, Mm00447665_m1; UP3A, Mm00447665_m1, GAPDH, Mm99999915_g1; OCT4, Mm00658129_gH; Nanog, Mm02019550_s1; REX1, Mm01194090_g1; α-actin, Mm00725412_s1; smooth muscle myosin heavy chain (SM-MHC), Mm00443013_m1; SM22α, Mm00441660_m1; human: α-actin, Hs00426835_g1; SM22α, Hs00162558_m1; GAPDH, Hs99999905_m1. For each cDNA sample, the threshold cycle (Ct) was defined as the cycle number at which amplification of the target gene was within the linear range of the reaction. Relative expression levels for each gene of interest were calculated by normalizing the target gene transcript level (Ct) to the respective GAPDH level with the maximum expression values per gene displayed at a 100 per condition as described previously [32].

### Histological and Immunohistochemical (IHC) Analyses

Non-seeded and seeded constructs were fixed in 10% neutral-buffered formalin, dehydrated in graded alcohols, and embedded in paraffin. For detection of fibronectin coatings, scaffolds were placed in OCT compound and frozen at -80°C. Serial sections (10 µm) were cut from paraffin and OCT blocks and either stained with hematoxylin and eosin (H&E) or subjected to IHC analyses. To verify ECM protein coatings on non-seeded scaffolds, the following primary antibodies were used: anti-collagen type I (Abcam, Cambridge, MA, cat.# ab34710, 1∶200 dilution), anti-collagen type IV (Abcam, cat.# ab6586, 1∶200 dilution), and anti-fibronectin (Millipore, cat.# AB2047, 1∶200 dilution). For differentiation analyses, contractile smooth muscle markers such as α-actin and SM22α [38] as well as urothelial-associated proteins, uroplakins (UP) [39] and cytokeratins (CK) [40], were detected using the following primary antibodies: anti-α-actin (Sigma-Aldrich, cat.# A2457, 1∶200 dilution), anti-SM22α (Abcam, Cambridge, MA, cat.# ab14106, 1∶200 dilution), anti-pan-UP (rabbit antisera raised against total bovine UP extracts, 1∶100 dilution), anti-pan-CK (Dako, Carpinteria, CA, cat.# M3515, 1∶200 dilution). Specimens were incubated with respective species-matched Cy3-conjugated secondary antibodies (Millipore) to ascertain primary antibody localization. Nuclei were counterstained with DAPI and sections were visualized using an Axioplan-2 microscope (Carl Zeiss MicroImaging, Thornwood, NY) and representative images were acquired using Axiovision software (version 4.8, Thornwood, NY).

### Statistical Analyses

All quantitative measurements were collected with N = 3–4 independent replicates per data point from one representative experiment and expressed as mean ± standard deviation. Data for these measurements were analyzed with the Mann-Whitney U test using SPSS Statistics software v19.0 (http://www.spss.com). Statistically significant values were defined as *p≤*0.05.

## Results and Discussion

**Figure 1:**
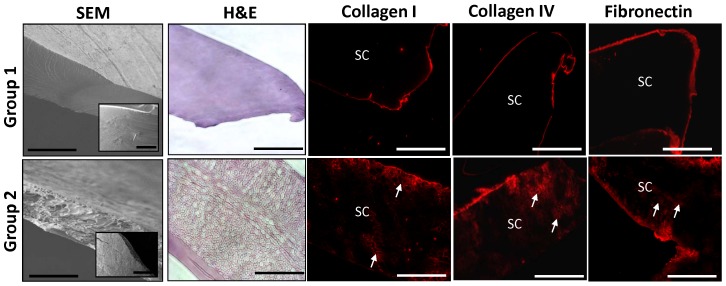
Structural morphology of silk scaffold groups and characterization of ECM coatings. SEM photomicrographs of cross-sectional and top views (inset) of silk scaffold configurations. Scale bars  =  300 µm; inset, scale bars  =  200 µm. Photomicrographs of H&E-stained cross-sections of unseeded scaffold groups and immunofluorescence of various ECM coatings (collagen types I or IV or fibronectin) on matrices prior to cell seeding with similar orientation (Cy3 fluorophore, red). Scale bars  =  100 µm. SC denotes scaffolds. Arrows denote intercalation of ECM coatings within scaffold pores.

We investigated two groups of silk scaffold configurations as potential templates for the development of cell-seeded constructs for bladder tissue engineering. These matrices were chosen based on their reported ability to support bladder augmentation as well as smooth muscle and urothelial regeneration in normal rodents [16], [17]. SEM analysis (Figure 1) demonstrated that Group 1 scaffolds consisted of compact multi-laminates of parallel-oriented silk fibers whose architecture was preserved following post-winding methanol treatment. In contrast, we observed that Group 2 scaffolds were composed of porous lamellar-like sheets buttressed by a dense outer layer, devoid of initial winding patterns, but with increased surface roughness compared to Group 1 (**Figure 1**). Comparisons between these structurally diverse scaffold groups allowed for an interrogation of how silk biomaterial morphology affects primary and pluripotent cell responses.

To more accurately mimic the natural tissue microenvironment, scaffolds in both groups were incubated with bladder-associated ECM components such as collagen types I or IV or fibronectin prior to cell seeding. In the normal bladder wall, collagen type I is primarily expressed in the lamina propria wherein it surrounds smooth muscle bundles and nerves [41]. The urothelium is supported by the basement membrane which is predominantly composed of a network of collagen type IV and laminin [41]. Fibronectin is a non-collagenous glycoprotein associated with superficial urothelial cells which also encompasses individual smooth muscle fibers and vessel walls within the lamina propria [41]. For each ECM investigated, IHC analyses demonstrated the formation of qualitatively similar degrees of protein coatings localized around the periphery of each scaffold group (**Figure 1**). In addition, Group 2 matrices also displayed intercalation of all ECM coatings within their internal porous network to similar degrees

**Figure 2:**
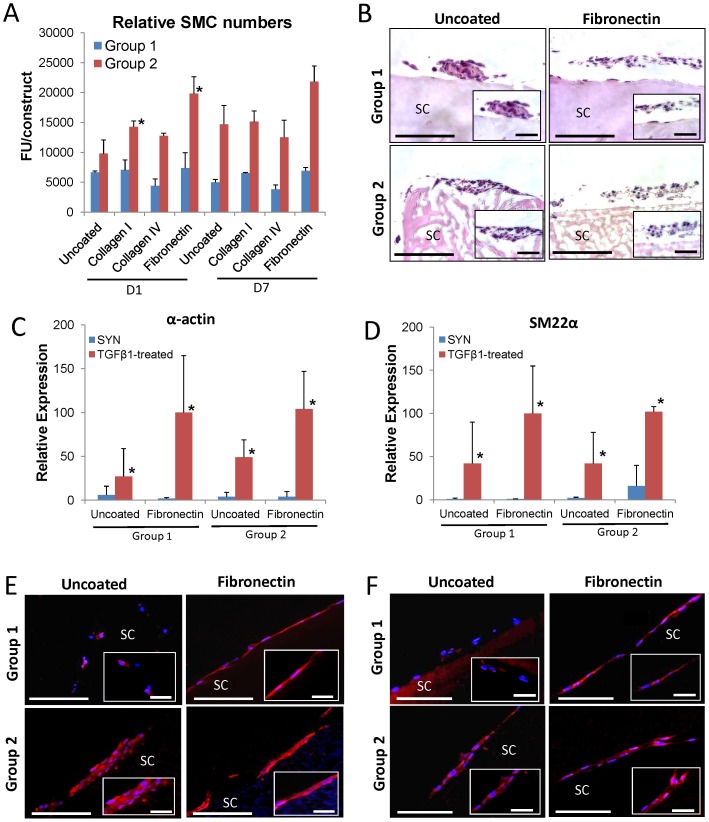
Silk biomaterial structure and specific ECM coatings influence SMC attachment and contractile differentiation. [**A**] AlamarBlue analysis of the extent of relative SMC attachment and proliferation on uncoated or ECM-coated (collagen types I or IV or fibronectin) silk groups following 1 and 7 d of culture, respectively. FU =  Fluorescent units, mean ± SD per data point. (*)  =  *p*≤0.05, in comparison to levels observed on uncoated Group 2 controls at 1 d. [**B**] Photomicrographs of SMC-seeded constructs (H&E-stained sections) following 7 d of culture as described in [A]. Scale bars  =  100 µm; magnified inset, scale bars  =  50 µm. [**C, D**] Real time RT-PCR analyses of mRNA transcript levels of contractile protein genes (α-actin [C] and SM22α [D]) in synthetic (SYN) and TGFβ1-treated constructs described in [A]. Levels normalized to GAPDH expression. Mean ± SD per data point. For each marker, (*)  =  *p*≤0.05, in comparison to levels in respective SYN controls. [**E, F**] Photomicrographs of contractile protein (α-actin [C] and SM22α [D]) expression in TGFβ1-treated constructs described in [C]. Immunofluorescence of contractile proteins (Cy3 fluorophore, red). DAPI nuclear counterstain (blue). Scale bars  =  100 µm; magnified inset, scale bars  =  50 µm. For [B, E, F], SC denotes scaffolds.

Uncoated and ECM-coated scaffold groups were assessed for their ability to support SMC attachment and proliferation (**Figure 2A, B**). Following 24 h of cell seeding, alamarBlue analysis demonstrated that uncoated Group 2 scaffolds supported elevated levels of relative cell attachment in comparison to uncoated Group 1 matrices. However, augmentation of Group 2 scaffolds with collagen type I or fibronectin significantly increased values over uncoated controls with the latter leading to the highest degree of cell attachment observed among any of the experimental groups at this timepoint. In contrast, ECM coatings had a negligible effect on the propensity of Group 1 scaffolds to support this parameter. Following 7 d of culture, no significant differences in relative cell numbers over day 1 levels were observed in any of the groups suggesting minimal cell proliferation. Histological comparisons of uncoated and fibronectin-coated groups at 7 d demonstrated that SMC were typically localized in patches of multi-cellular layers dispersed around the periphery of each scaffold group (**Figure 2B**), despite the increased surface area present within the porous network of Group 2 scaffolds. These results suggest that elevated alamarBlue levels seen in SMC cultured on Group 2 matrices in comparison to Group 1 may be a consequence of differences in other structural features between the groups. One probable candidate is the increased level of surface roughness encountered on Group 2 scaffolds. Indeed, a previous study of vascular SMC responses to metallic stents showed a positive correlation between the extent of cell adhesion and the degree of surface roughness [**42**]. These data collectively show that the structural architecture of Group 2 scaffolds coupled with fibronectin coatings produced a superior substrate for promoting SMC attachment in comparison to other scaffold formulations tested.

Following tissue isolation and *ex vivo* expansion, SMC derived from both vascular and visceral organs dedifferentiate from a quiescent contractile phenotype into a proliferative, synthetic state [43], [44]. In order for tissue engineered constructs to reconstitute the contractile properties of bladder defects, biomaterial configurations must support contractile differentiation of *ex vivo* expanded, synthetic SMC. Previous studies have demonstrated the ability of serum deprivation in combination with TGF-β1 treatment to stimulate contractile differentiation of ureteral SMC [43]. Therefore, we investigated the potential of these differentiation stimuli to elicit similar responses in bladder SMC cultured on uncoated and fibronectin-coated silk scaffolds. Real time RT-PCR analyses demonstrated significant upregulation of contractile marker expression including α-actin and SM22α in TGFβ1-treated SMC cultured on all matrices examined (**Figure 2C, D**). However, fibronectin-coated constructs in each group displayed the highest degree of expression of both contractile genes in comparison to their uncoated counterparts. IHC analyses revealed similar degrees of α-actin and SM22α protein expression in TGFβ1-treated SMC cultured on both fibronectin-coated groups (**Figure 2E, F**). In addition, cells stimulated on uncoated Group 2 scaffolds exhibited comparable levels of α-actin and SM22α expression in respect to fibronectin-coated constructs; however SMC cultured on uncoated Group 1 scaffolds displayed weak and sparse expression of both markers. Overall these results demonstrate that silk groups modified with fibronectin coatings are efficacious in supporting SMC contractile differentiation.

**Figure 3:**
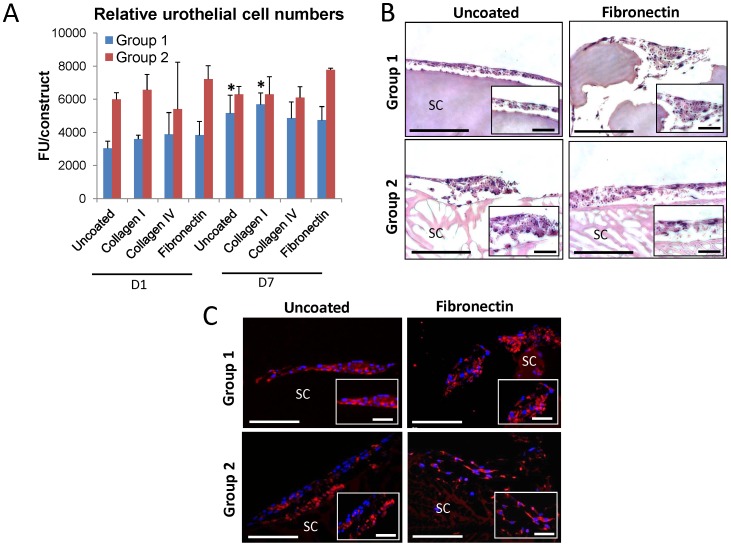
Urothelial cell attachment and proliferation are modulated by silk biomaterial morphology and selective ECM coatings. [A] AlamarBlue analysis of the extent of relative urothelial cell attachment and proliferation on uncoated or ECM-coated (collagen types I or IV or fibronectin) silk groups following 1 and 7 d of culture, respectively. FU =  Fluorescent units, mean ± SD per data point. (*)  =  *p*≤0.05, in comparison to levels observed on respective 1 d counterparts. [B] Photomicrographs of urothelial cell-seeded constructs (H&E-stained sections) following 7 d as described in [A]. Scale bar  =  100 µm; inset, scale bar  =  50 µm. [C] Photomicrographs of pan-cytokeratin protein expression in urothelial cells cultured for 9 d on control and fibronectin-coated groups. Immunofluorescence of pan-cytokeratins (Cy3 fluorophore, red). DAPI nuclear counterstain (blue). Scale bars  =  100 µm; magnified inset, scale bars  =  50 µm. For [B,C], SC denotes scaffolds.

The bladder and associated urinary tract are lined by the urothelium, a transitional epithelium which acts as a specialized permeability barrier that protects the underlying tissue from toxic urinary components [39]. The ability of tissue-engineered constructs to reconstitute the barrier function of bladder defects depends on the potential of scaffold configurations to support urothelial integration and differentiation. Uncoated and ECM-coated silk matrices were evaluated for their capacity to promote urothelial cell attachment and proliferation (**Figure 3A, B**). AlamarBlue analysis revealed that following initial cell seeding, uncoated Group 2 scaffolds supported substantially higher degrees of relative cell attachment in comparison to uncoated Group 1 matrices. In addition, fibronectin coating of Group 2 scaffolds further increased the extent of cell attachment to levels higher than observed with any other ECM coating. However, relative urothelial cell attachment to Group 1 matrices was minimally effected by the presence of any of the ECM coatings tested.

Over the course of 7 d of cultivation, relative cell numbers significantly increased on uncoated and collagen I-coated Group 1 matrices in comparison to day 1 levels. In contrast, minimal urothelial cell proliferation occurred on uncoated or ECM-coated Group 2 scaffolds. However, fibronectin-coated Group 2 matrices maintained the highest level of relative urothelial cell numbers in comparison to all other experimental conditions tested at 7 d. Histological comparisons of uncoated and fibronectin-coated groups at this timepoint demonstrated dispersed regions of multi-cellular layers of urothelial cells primarily lining the periphery of all scaffold configurations. Similar to the responses seen in SMC, the overall higher degrees of cellularity observed in Group 2 scaffolds, as determined by alamarBlue analysis, may be due to increased surface roughness encountered on this matrix configuration. A study by Chun and colleagues provided evidence for this notion showing increased urothelial cell adhesion to poly-lactic-co-glycolic acid and poly-(ether)-urethane polymers following enhancement of surface roughness by chemical etching [45]. IHC analyses confirmed the ability of uncoated and fibronectin-coated scaffold groups to support expression of differentiation-associated markers such as cytokeratins in seeded urothelial cells by demonstrating prominent protein staining following 9 d of culture (**Figure 3C**). Overall, these findings show that fibronectin-coated Group 2 scaffolds provide an optimal scaffold for urothelial cell responses in comparison to other matrix configurations by promoting the highest degree of construct cellularity while supporting a differentiated phenotype.

**Figure 4:**
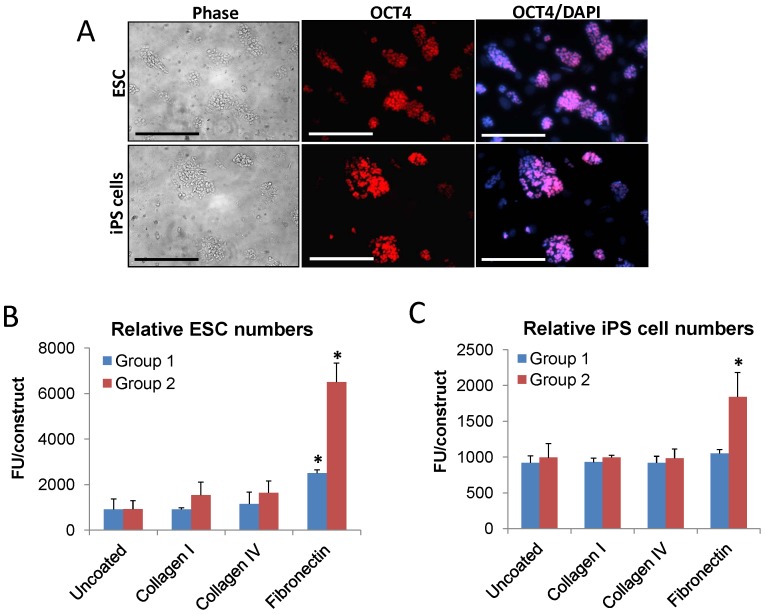
Group 2 silk scaffold architecture coupled with fibronectin coatings maximizes pluripotent stem cell attachment. [**A**] Photomicrographs of phase contrast images of naïve ESC and iPS cells prior to scaffold seeding. Immunofluorescence of the pluripotent marker, OCT4, in ESC and iPS cells (Cy3 fluorophore, red). DAPI nuclear counterstain (blue). Scale bars for phase and immunofluorescence images  =  100 µm. [**B, C**] AlamarBlue analysis of the extent of relative ESC [B] and iPS cell [C] attachment on uncoated or ECM-coated (collagen types I or IV or fibronectin) silk groups following 1 d of culture. FU =  Fluorescent units, mean ± SD per data point. (*)  =  *p*≤0.05, in comparison to levels observed on respective uncoated controls.

ESC and iPS cells were investigated for their potential to function as cell sources for bladder tissue engineering. Phase contrast microscopy and ICC analyses of ESC and iPS cell lines revealed similar morphological features including the formation of refractile, multi-cellular colonies during *ex vivo* expansion which exhibited prominent nuclear expression of the pluripotency marker, OCT4, in contrast to MEF feeder layers (**Figure 4A**). The ability of uncoated and ECM-coated scaffolds to support ESC and iPS cell attachment was determined by AlamarBlue analysis (**Figures 4B, C**). For each respective cell line, similar degrees of relative cell attachment were observed between Group 1 and 2 scaffolds either uncoated or coated with collagen types I or IV. In contrast, fibronectin coatings significantly increased the degree of ESC attachment on both types of matrix configurations in respect to uncoated controls with Group 2 matrices displaying the highest levels over all conditions. Fibronectin coatings did not affect the propensity of Group 1 scaffolds to promote iPS cell attachment over control levels; however this ECM coating did significantly elevate the extent of cell attachment over uncoated matrices. These data demonstrate that the combination of matrix architecture and the presence of fibronectin coatings play a major role in influencing the extent of pluripotent cell attachment.

**Figure 5:**
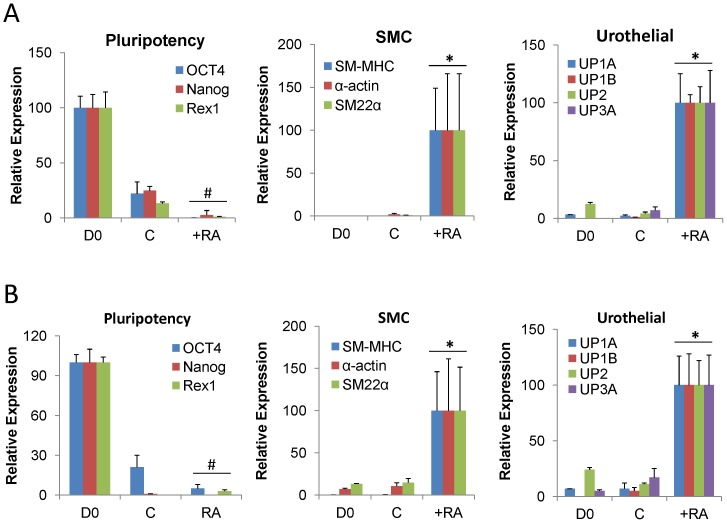
RA stimulates SMC and urothelial markers in pluripotent stem cells cultured on silk scaffolds. [**A, B**] Real time RT-PCR analyses of mRNA transcript levels of pluripotency transcription factors (OCT4, Nanog, REX1), SMC contractile genes (SM-MHC, α-actin, SM22α), and urothelial-associated uroplakins (UP) in ESC [A] or iPS cells [B] cultured on fibronectin-coated Group 2 matrices for 14 d in the presence of RA or maintained as spontaneously differentiating controls (C). D0  =  naïve, undifferentiated controls. Levels normalized to GAPDH expression. Mean ± SD per data point. (#)  =  *p*≤0.05, in comparison to D0. (*)  =  *p*≤0.05, in comparison to D0 and C conditions.

**Figure 6:**
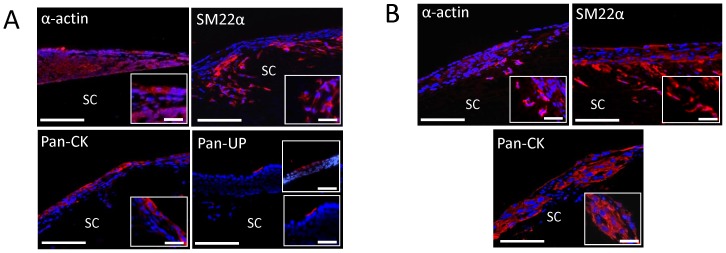
SMC and urothelial populations in RA-treated pluripotent stem cells cultured on silk scaffolds. [**A, B**] Photomicrographs of SMC contractile protein expression (α-actin and SM22α) and urothelial-associated markers [pan-cytokeratins (CK) and pan-uroplakins (UP)] in ESC [A] and iPS cells [B] cultured on fibronectin-coated Group 2 matrices for 14 d in the presence of RA. (Cy3 fluorophore, red). DAPI nuclear counterstain (blue). Scale bars  =  100 µm; magnified inset, scale bars  =  50 µm. SC denotes scaffolds in all panels.

RA is known to function in both a time- and concentration-dependent manner to selectively regulate the specification of ESC and iPS cells into various types of differentiated lineages [38], [46], [47]. In particular, our previous study demonstrated the ability of continuous stimulation of ESC with micromolar concentrations of RA to promote both urothelial and SMC differentiation in monolayer cultures as well as neuronal and extra-embryonic cell types [32]. Although RA stimulation has been reported to induce SMC differentiation in iPS cells [33], its ability to stimulate urothelial differentiation is unknown. Therefore, we hypothesized that fibronectin-coated Group 2 matrices would function as biocompatible substrates to support RA-mediated differentiation of both ESC and iPS cells toward SMC and urothelial lineages. Over the course of the 14 d culture period, real time RT-PCR analyses demonstrated that in response to RA, ESC (**Figure 5A**) and iPS cells (**Figure 5B**) elicited significant downregulation of all pluripotency factor mRNA transcript levels tested in comparison to naïve controls. In addition, RA stimulation promoted significant induction of all urothelial-associated uroplakin and smooth muscle contractile gene mRNA transcript levels tested in comparison to undifferentiated and spontaneously differentiating controls. Parallel IHC analyses of RA-treated constructs seeded with ESC (**Figure 6A**) and iPS cells (**Figure 6B**) demonstrated the formation of distinct cell populations with fibroblastic morphology displaying prominent contractile protein expression, including α-actin and SM22α, indicative of SMC differentiation. In addition, cytokeratin-positive cells with cuboidal epithelial-like morphology were also detected in both seeded cell populations in response to RA. Urothelial maturation of RA-treated ESC was evident in epithelial subpopulations which exhibited uroplakin protein expression. In contrast, uroplakin-positive cells were not detected in iPS cell-seeded constructs cultured under similar conditions. These results provide evidence that silk scaffolds are permissive for SMC and urothelial differentiation of pluripotent cell lines. However, ESC may be a more optimal cell source for bladder tissue engineering given the higher degree of urothelial maturation observed in comparison to iPS cells.

## Conclusion

Structural morphology and the presence of specific ECM coatings were found to be significant factors in influencing the performance of silk biomaterials to support primary and pluripotent cell attachment and differentiation. In comparison to other scaffold formulations evaluated in this study, the combination of fibronectin coatings with Group 2 scaffold architecture promoted the highest level of primary SMC and urothelial cell attachment as well as supported their respective differentiated phenotypes. In addition, this matrix was also superior in mediating ESC and iPS cell attachment and upon treatment with RA, SMC and urothelial differentiation could be achieved. Previous reports have demonstrated the ability of conventional scaffolds such as PGA and SIS to support primary SMC and urothelial attachment and differentiation [4], [7], however fibronectin-coated Group 2 silk matrices offer the additional advantage of supporting maturation of pluripotent cell types into bladder-associated lineages which may be useful in bladder reconstruction strategies when primary cell function is compromised due to disease. In summary, fibronectin-coated Group 2 scaffolds consisting of rough, porous lamellar-like sheets of gel spun silk fibroin represent promising platforms for cell-seeded bladder tissue engineering approaches.
